# Supplementary material for: Chromatin compaction precedes apoptosis in developing neurons
Source: Commun Biol. 2022 Aug 8;5:797. doi: 10.1038/s42003-022-03704-2 (PMC9359995; doi:10.1038/s42003-022-03704-2)
Supplement: Supplementary file 2 — Supplementary Information [file 42003_2022_3704_MOESM2_ESM.pdf]

Supplemental information

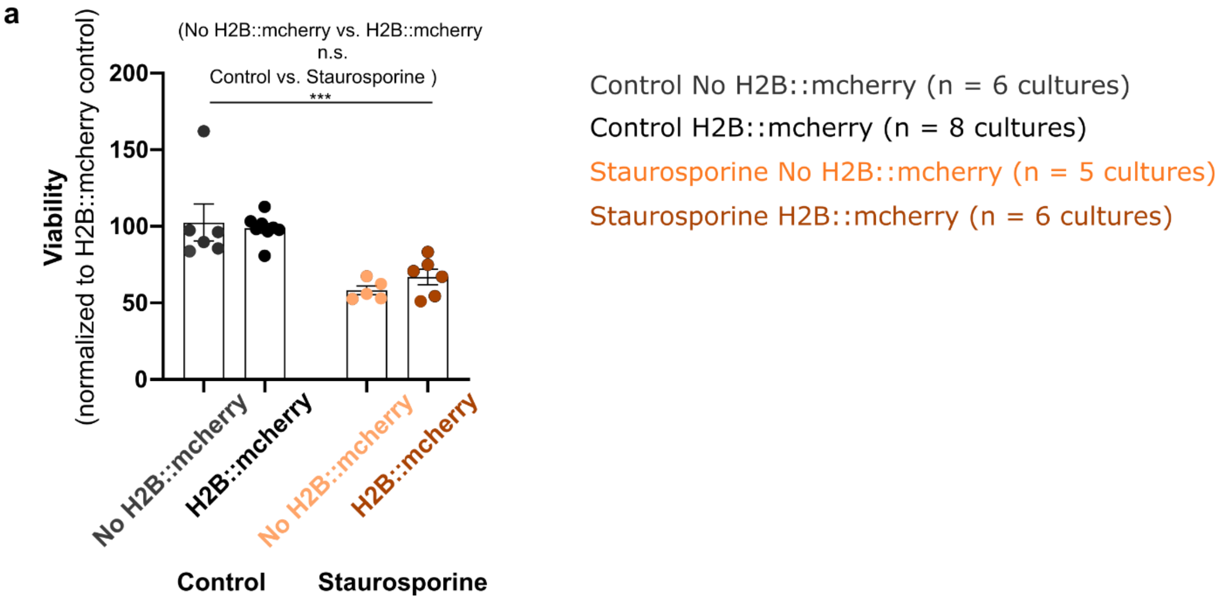

Supplemental Figure 1. Normal viability of primary cortical neurons with H2B::mCherry overexpression under control conditions and no difference in viability under staurosporine-treatment compared to neurons without H2B::mCherry. a) Alamar-based assay did not show a significant difference in viability when comparing viability of No H2B::mCherry vs H2B::mCherry neurons. 4h treatment with staurosporine (1.5µM) resulted in a similar reduction of neuronal viability. Data are represented as mean ± SEM. Two-way ANOVA was applied for comparison of differences between control and staurosporine F (1, 21) = 31,34 p<0.0001 and No H2B::mCherry vs. H2B::mCherry F (1, 21) = 0,1335 and p=0.72.

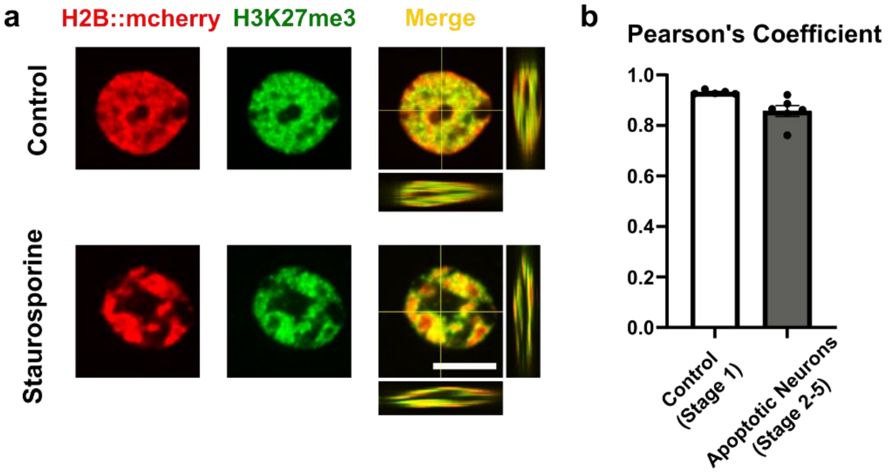

Supplemental Figure 2. Co-localization of heterochromatin marker H3K27me3 with H2B::mCherry fluorescence signal. a) Representative confocal images of nuclei of a neuron under control condition and upon induction of apoptosis with staurosporine (scale 5µm). **b)** High average Pearson's Coefficients show strong correlation of immunohistochemical signal of anti-H3K27me3 staining with the transgenic H2B::mCherry signal under control conditions (later defined as stage 1, n=5 cells) and during all phases of apoptosis (stage 2-5, n=7 cells).

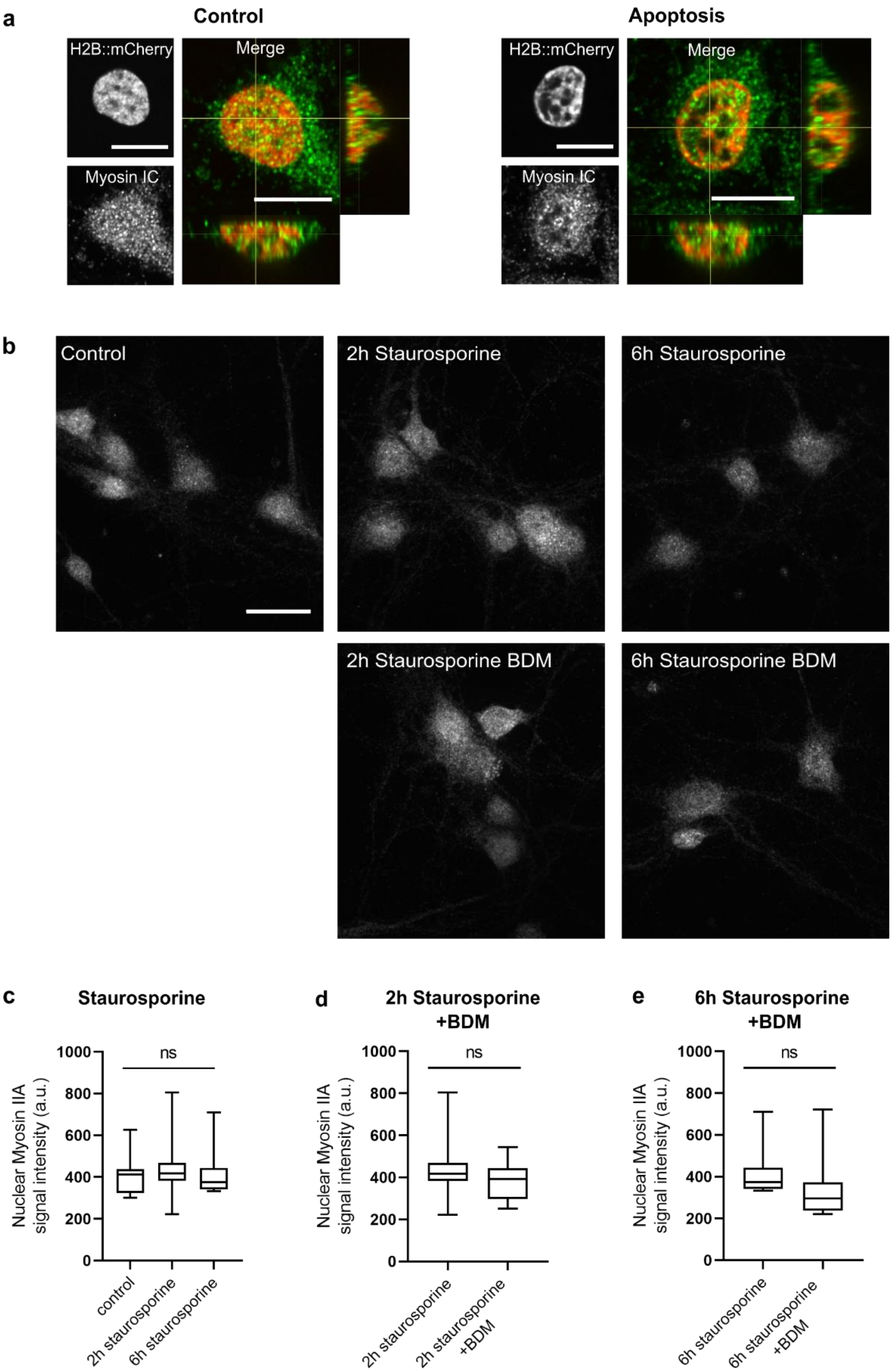
a
Control
Apoptosis
H2B::mCherry
Myosin IC
Merge
b
Control
2h Staurosporine
6h Staurosporine
2h Staurosporine BDM
6h Staurosporine BDM
c
Staurosporine
d
2h Staurosporine +BDM
e
6h Staurosporine +BDM
Nuclear Myosin IIA signal intensity (a.u.)
ns
1000
800
600
400
200
0
control
2h staurosporine
6h staurosporine
2h staurosporine +BDM
6h staurosporine +BDM

Supplementary Figure 3. Myosin IIA is expressed in the nucleus of primary cortical neurons at similar levels under control and apoptotic conditions and nuclear expression levels are not affected by the additional application of BDM. a) Confocal images of immunostainings against myosin IIA and H2B::mCherry signal confirm the expression of myosin IIA in the nucleus of cortical neurons both under control conditions and in apoptotic neurons (scale 10µm). **b)** Representative average z-stack projections of myosin IIA signals under untreated control conditions, 2h and 6h after of staurosporine application and upon additional treatment with the actomyosin inhibitor BDM (scale 20µm). **c)** Nuclear myosin IIA signal intensity was not significantly altered by application of staurosporine (n=13/11/11 cells). **d,e)** The additional application of BDM did not affect the nuclear myosin IIA signal intensity neither after 2h nor 6h (n = 11 cells per condition). Data are represented as boxplots, whiskers MIN to MAX. One-way ANOVA was applied for comparison of differences between control and staurosporine 2h and 6h $F(2, 32) = 0.37$ $p=0.70$ and t-test for comparison of nuclear myosin IIA signal intensity upon application of staurosporine only with staurosporine plus BDM after 2h ($p=0.24$), and 6h ($p=0.10$).

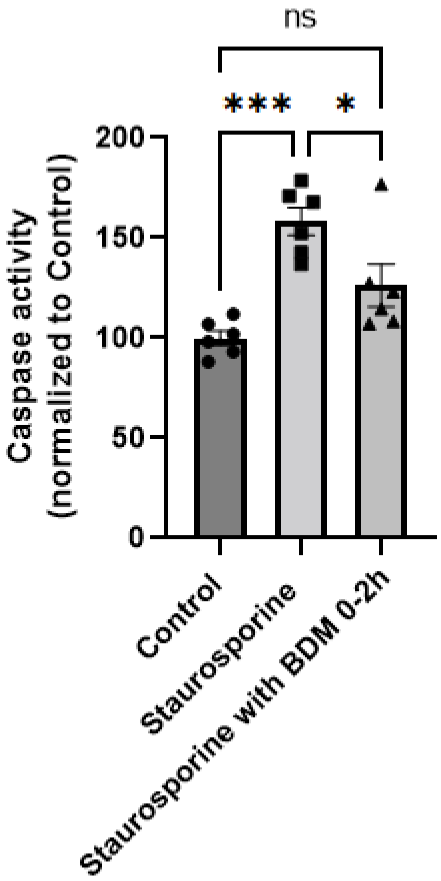

Supplementary Figure 4. The presence of BDM during the early phase (0-2h) mitigates the staurosporine-induced caspase activation after 6h. Caspase activity measured by luminescent caspase assay at DIV8 or DIV9 under untreated control conditions, as well as upon staurosporine treatment for 6h as well as staurosprine treatment for 6h with BDM application from 0-2h. Data are represented as mean ± SEM. n=6 cultures per condition. One-way ANOVA was applied to detect differences across pharmacological treatments $F(2,15)=14.78$ and $p=0.0003$ and results from subsequent Tukey´s multiple comparison test are shown.

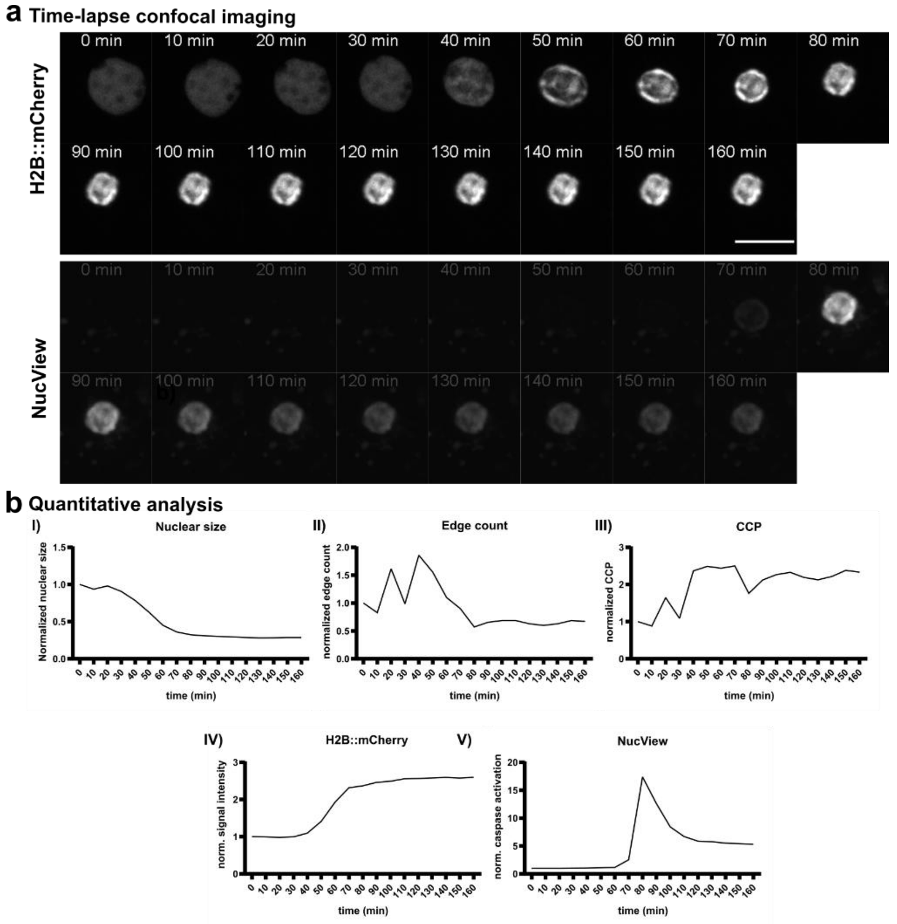

Supplementary Figure 5. High-resolution, confocal time-lapse imaging (a) and quantitative analysis (b) of a single, representative neuronal nucleus before, during and after apoptosis confirms that the increase in chromatin compaction precedes the decrease in nuclear size and caspase activation. Confocal images were acquired every 10min, starting before the apoptosis process began until the end of apoptosis. Already before nuclear shrinkage (I, from 40 min onwards), chromatin becomes more compacted throughout apoptosis, i.e. increase in Edge count (II) and CCP (III) starting to rise with 20 min. In line with the continuous shrinkage of the nucleus, the H2B::mCherry signal becomes more intense from 40-50 min on (IV). Caspase activation, visualized by the NucView signal (V) follows with a delay in time at around 70-80 min, where after it slowly fades.
