# Supplementary material for: Chromatin compaction precedes apoptosis in developing neurons
Source: Commun Biol. 2022 Aug 8;5:797. doi: 10.1038/s42003-022-03704-2 (PMC9359995; doi:10.1038/s42003-022-03704-2)
Supplement: Supplementary file 3 — Description of Additional Supplementary Files [file 42003_2022_3704_MOESM3_ESM.pdf]

Description of Additional Supplementary Files

File name: Supplementary movie 1
Description: Representative video showing dynamics of nuclear chromatin (H2B::mCherry signal) of a primary cortical neuron at DIV 8 under control conditions. Images were acquired every 10min for 420 min (30min baseline +380min treatment). Scale: 10µm, images displayed at 3fps.

File name: Supplementary movie 2
Description: : Representative video showing dynamics of chromatin (H2B::mCherry signal) of a primary cortical neuron at DIV 8 undergoing spontaneous apoptosis. Note, early chromatin compaction (i.e. granulation) that precedes cell shrinkage and final nuclear condensation.

File name: Supplementary movie 3
Description: Representative Representative video showing dynamics of chromatin (H2B::mCherry signal) of a primary cortical neuron at DIV 8 undergoing staurosporine-induced apoptosis. High resolution confocal images were acquired every 10min, for 30min-baseline acquisition, and for 380min in presence of staurosporine (1.5µM). Note, early chromatin compaction (i.e. granulation) that precedes cell shrinkage, nuclear fragmentation and condensation.

File name: Supplementary movie 4
Description: Representative video showing dynamics of chromatin (H2B::mCherry signal) of a primary cortical neuron at DIV 8 undergoing staurosporine-induced apoptosis. High resolution confocal images were acquired every 10min, for 30min-baseline acquisition, and for 380min in presence of staurosporine (1.5µM). Also here, please note early chromatin compaction (i.e. granulation) that precedes cell shrinkage and condensation.

File name: Supplementary movie 5
Description: Representative video showing dynamics of chromatin (H2B::mCherry signal) of a primary cortical neuron at DIV 8 undergoing non-apoptosis, necrosis-like cell death. Images were acquired every 10min for 420 min (30min baseline +380min). Note, nuclear swelling that precedes final nuclear shrinkage and concurrent compaction of chromatin.
